# Time To Move from Presumptive Malaria Treatment to Laboratory-Confirmed Diagnosis and Treatment in African Children with Fever

**DOI:** 10.1371/journal.pmed.0050252

**Published:** 2009-01-06

**Authors:** Valérie D'Acremont, Christian Lengeler, Hassan Mshinda, Deo Mtasiwa, Marcel Tanner, Blaise Genton

## Abstract

Background to the debate: Current guidelines recommend that all fever episodes in African children be treated presumptively with antimalarial drugs. But declining malarial transmission in parts of sub-Saharan Africa, declining proportions of fevers due to malaria, and the availability of rapid diagnostic tests mean it may be time for this policy to change. This debate examines whether enough evidence exists to support abandoning presumptive treatment and whether African health systems have the capacity to support a shift toward laboratory-confirmed rather than presumptive diagnosis and treatment of malaria in children under five.

## Abstract 2

Blaise Genton and colleagues argue that we should abandon presumptive antimalarial treatment in children with fever in sub-Saharan Africa, while in a related paper Mike English and colleagues disagree that we have sufficient evidence and health system capacity to do so.

*In this Viewpoint, Blaise Genton and colleagues argue in favor of abandoning presumptive treatment for under-fives. Mike English and colleagues present the opposing Viewpoint in a related article: English M, Reyburn H, Goodman C, Snow RW (2009) Abandoning presumptive antimalarial treatment for febrile children aged less than five years—A case of running before we can walk? PLoS Med 6(1): e1000015. doi: 10.1371/journal.pmed.1000015*

Malaria has long been the number one cause of fever and the leading cause of child mortality in sub-Saharan Africa. As a result, the World Health Organization (WHO) recommends treating any fever episode in African children with antimalarial drugs to save lives. However, this approach may not be as safe as it was 20 years ago for two major reasons. Firstly, the proportion of fevers due to malaria has become significantly lower, even in highly endemic areas, and hence the relative likelihood of missing other potentially fatal diseases has become higher. Secondly, we now have new reliable rapid diagnostic tests (RDTs) to allow proper diagnosis of malaria at all levels of the health system.

## Evidence of Decreased Malaria Transmission in Sub-Saharan Africa, and Declining Proportion of Fevers Due To Malaria

There is growing evidence documenting a substantial decline in malaria transmission, morbidity, and mortality in more than 13 African countries where malaria control interventions have been implemented at scale. This reduction is also observed in areas with previously high levels of transmission (e.g., [1]). Although available data are not always spatially congruent, and therefore cannot necessarily be viewed as representing secular changes, the sharp decline in sub-Saharan Africa of the Plasmodium falciparum prevalence rate in children aged two to ten years—from 37% in the years 1985–1999 to 17% in 2000–2007—clearly documents this trend [2]. This decrease implies that many of the areas previously defined as “high stable malaria transmission” have changed, or will soon change, into “moderate to low transmission” areas.

The lower the transmission, the lower the probability that a fever episode will be due to malaria. In Tanzania, a high-endemicity country, only one to four out of ten under-five patients with fever are parasitaemic in the rural settings (A. M. Kabanywany, 2007 and V. D'Acremont, 2008, personal communications) [3]. With a decline in malaria's prevalence, the hazard of misdiagnosis of many children becomes significant. When giving an antimalarial, the health worker is less likely to look for another treatable cause of fever, and this leads to higher morbidity and mortality due to delay in giving appropriate treatment, as suggested by studies that showed higher case fatality rates among non-malaria fevers compared to malaria fevers [4].

## Availability of RDTs for Malaria

The shift from symptom-based diagnosis to parasite-based management of malaria requires that clinicians have a reliable, easy-to-use, and inexpensive diagnostic test. It should have good sensitivity, require minimal training and equipment, and retain accuracy even after extensive storage under tropical conditions. All these characteristics are met by the new generation of RDTs. Two meta-analyses have clearly shown that the performance of RDTs is comparable to that of expert microscopy [5,6]. Appropriate action taken on the basis of the test result is now the key element for the successful introduction of RDTs. Unfortunately, this has not always been achieved in the implementation of RDTs so far, partly because the training was insufficient to change the clinician's perception of malaria risk [7]. In addition, the ambiguous messages from WHO and national guidelines stating that malaria should be considered even in the presence of a negative test have added to the confusion [8].

At present risk levels, the risk of missing a malaria case due to a false-negative test is substantially smaller than the risk of the patient dying due to another severe disease because of the focus on malaria. The risk of a false-negative test and its potential consequences have recently been evaluated thoroughly in Uganda (using microscopy) [9] and in Tanzania (using RDTs) [10], and the safety of not treating malaria-negative children confirmed.

Other compelling arguments for systematic testing are listed in Box 1 [11,12].

**Box 1. Additional Arguments for a Shift from Presumptive Malaria Treatment to Laboratory-Confirmed Diagnosis and Treatment According To Test Results in Children Under Five with Fever**

- Treating all fever patients with antimalarials leads to a huge drug wastage, and hence potential for drug shortage
- Inappropriate use of antimalarials leads to unnecessary adverse drug reactions
- Irrational use of antimalarial drugs leads to increased parasite resistance
- Potential mistrust on the part of the public on the real efficacy of artemisinin-based combination therapies (ACTs) due to use for inappropriate indications (viral or bacterial disease)
- Parasitological diagnosis and treatment with ACTs according to test results versus presumptive treatment with ACTs is costeffective in all current malaria-endemic situations (as long as test result is taken into account) [11,12]

## Caveat

A switch from presumptive treatment to laboratory-confirmed diagnosis and treatment is now urgent but needs to be carefully planned. Large-scale deployment of RDTs is a great challenge that requires theoretical and practical training, regular supervision, and sustained financial mechanisms to ensure constant availability. It is crucial that quality control is implemented at all steps.

Also, introduction of reliable diagnosis implies that clinicians need to be trained to manage the “negative syndrome” (patients with a negative malaria test). This is challenging after years of upholding the notion that fever equals malaria and requires substantial change in the behaviour of clinicians and caretakers. This is now a great opportunity to update and re-strengthen the Integrated Management of Childhood Illnesses to promote improved case management of African children.

## Conclusion

The recent trend of malaria decline in Africa calls for a shift from presumptive treatment to laboratory-confirmed diagnosis and treatment in all areas, regardless of age and level of malaria transmission. Such a move is especially relevant with the new momentum towards elimination and is now realistic thanks to reliable RDTs. As part of renewed malaria control efforts, it is time to improve clinical management and abandon irrational use of drugs, i.e., antimalarial treatment for no malaria and no treatment for other potentially fatal causes of fevers.

## Genton and Colleagues' Response to English and Colleagues' Viewpoint

*In this section, Genton and colleagues respond to the points raised by English and colleagues in their opposing Viewpoint: English M, Reyburn H, Goodman C, Snow RW (2009) Abandoning presumptive antimalarial treatment for febrile children aged less than five years—A case of running before we can walk? PLoS Med 6(1): e1000015. doi: 10.1371/journal.pmed.1000015*

We all agree that laboratory-confirmed diagnosis of malaria is the desirable goal. We disagree on the present status of evidence and the conditions needed to change the policy.

## Evidence on RDT Performance

Evidence is accumulating showing that RDT performance is high, even when used in routine practice [13–15]. The authors of the study showing 65% sensitivity acknowledge themselves that “use of poor quality blood smear may impede reliable measurement of sensitivity and specificity and undermine confidence in the new diagnostic” [16]. The issue nowadays is no longer about RDT performance but about how to best implement quality assurance.

## Evidence on the Safety of the Management Strategy Based on Laboratory Confirmation of Parasites

Although the debate about laboratory-confirmed diagnosis versus presumptive treatment has been ongoing for years, nobody has embarked on the definitive study to show that mortality is not higher with the former than the latter. We have recent evidence confirming that the strategy based on documented diagnosis is safe [9], even in uncontrolled settings [10]. We consider that accumulated experience in all age groups and studies including more than 3,000 children under five are enough to decide on a policy change.

## Evidence on Health Workers' Beliefs and Behaviour

New evidence shows that clinicians trust and act upon RDT results. Indeed, in recent studies conducted under programmatic conditions, 2%–10% of all patients with negative results were given antimalarials [16] (D'Acremont et al., unpublished data). This contrasts with disappointing results published earlier [17] and illustrates the behaviour change that can be achieved through proper training and trust gained by accumulating field experience with RDTs.

To align practices in formal care settings with those at community level, we propose to also use RDTs outside health facilities. It is safe and feasible [18]. Moreover, it fits with the new WHO Special Programme for Research and Training in Tropical Diseases and UNICEF initiative that promotes community-integrated management of malaria/pneumonia/diarrhoea rather than home-based management of malaria.

## More Data on Malaria Epidemiology

Improved data on local malaria epidemiology will not help much. We already know that heterogeneity is huge and rapidly evolving. Only a uniform policy across all age groups and epidemiological settings is likely to be implemented appropriately. This is best illustrated by the conclusion made by one of the authors of the opposing Viewpoint in a recent paper: “Despite different recommendations for patients below and above 5 years of age, malaria diagnosis and treatment practices were similar in the two age groups… Malaria diagnosis recommendations differing between age groups appear complex to implement; further strengthening of diagnosis and treatment practices under AL policy is required.” [19].

## Conclusion

We strongly believe that the policy should be changed even if all conditions for perfect implementation are not fulfilled. In areas where RDTs have been deployed at scale, clinicians have understood that the performance and usefulness of RDTs is not different in a child under five years than in an adult. The time needed to achieve all conditions English et al. consider necessary to have a policy change will only lead to more deaths due to diseases other than malaria left untreated and to the emergence of parasite resistance consequent to irrational use of drugs. A policy change is desirable to deliver clear and simple messages. Staggered implementation will follow according to country capacity, as is done for laboratory-confirmed diagnosis and treatment in other age groups.

## Abbreviations

ACT: artemisinin-based combination therapy
RDT: rapid diagnostic test
WHO: World Health Organization
